# Comparison of Compound Identification Tools Using Data Dependent and Data Independent High-Resolution Mass Spectrometry Spectra

**DOI:** 10.3390/metabo13070777

**Published:** 2023-06-21

**Authors:** Rosalie Nijssen, Marco H. Blokland, Robin S. Wegh, Erik de Lange, Stefan P. J. van Leeuwen, Bjorn J. A. Berendsen, Milou G. M. van de Schans

**Affiliations:** Wageningen Food Safety Research, Part of Wageningen University and Research, Akkermaalsbos 2, 6708 WB Wageningen, The Netherlands

**Keywords:** identification, annotation, pesticides, veterinary drugs, metabolites, data independent acquisition, data dependent acquisition, high-resolution mass spectrometry

## Abstract

Liquid chromatography combined with high-resolution mass spectrometry (LC-HRMS) is a frequently applied technique for suspect screening (SS) and non-target screening (NTS) in metabolomics and environmental toxicology. However, correctly identifying compounds based on SS or NTS approaches remains challenging, especially when using data-independent acquisition (DIA). This study assessed the performance of four HRMS-spectra identification tools to annotate in-house generated data-dependent acquisition (DDA) and DIA HRMS spectra of 32 pesticides, veterinary drugs, and their metabolites. The identification tools were challenged with a diversity of compounds, including isomeric compounds. The identification power was evaluated in solvent standards and spiked feed extract. In DDA spectra, the mass spectral library mzCloud provided the highest success rate, with 84% and 88% of the compounds correctly identified in the top three in solvent standard and spiked feed extract, respectively. The in silico tools MSfinder, CFM-ID, and Chemdistiller also performed well in DDA data, with identification success rates above 75% for both solvent standard and spiked feed extract. MSfinder provided the highest identification success rates using DIA spectra with 72% and 75% (solvent standard and spiked feed extract, respectively), and CFM-ID performed almost similarly in solvent standard and slightly less in spiked feed extract (72% and 63%). The identification success rates for Chemdistiller (66% and 38%) and mzCloud (66% and 31%) were lower, especially in spiked feed extract. The difference in success rates between DDA and DIA is most likely caused by the higher complexity of the DIA spectra, making direct spectral matching more complex. However, this study demonstrates that DIA spectra can be used for compound annotation in certain software tools, although the success rate is lower than for DDA spectra.

## 1. Introduction

Liquid chromatography combined with high-resolution mass spectrometry (LC-HRMS) is a frequently applied technique for suspect screening (SS) and non-target screening (NTS) in metabolomics, human biomonitoring, and environmental toxicology [1]. However, correctly identifying compounds based on SS or NTS approaches remains challenging [2,3,4,5]. In most studies, only a small percentage of compounds are identified. The molecular formula of unknown compounds detected in LC-HRMS data can be derived from MS^1^ data by using the accurate mass and isotope pattern and applying the seven golden rules [6]. For further compound identification, MS^2^ spectra are preferred. A reference standard is needed for compound identification, and both the spectrum and retention time must match the reference standard. A lack of reference standards was recently identified [7] as a major challenge in environmental and human risk assessment sciences, calling—among other measures—for better availability of reference standards. Putative identification of compounds is often performed because of the limited availability of reference standards. For putative identification, the MS^2^ spectrum must match a spectrum in a mass spectral library or diagnostic evidence, such as characteristic fragments, as Schymanski et al. described [8].

Mass spectral libraries containing experimental MS^2^ spectra for compound identification are continuously growing. MzCloud [9] and Massbank of North America (MoNA) are two examples of mass spectral libraries. mzCloud contains over 9 million HR-MS spectra obtained with orbitrap instruments, covering over 20,000 unique compounds, and is commercially maintained. MoNA includes almost 2 million spectra, a combination of both user-generated HRMS spectra obtained with (Q)-TOF or orbitrap instruments and in silico predicted spectra, covering over 650,000 unique compounds. Note that mass spectral libraries only cover a limited number of compounds; the CAS registry contains over 127 million unique compounds. Mass spectral libraries are growing, but their growth is limited by the costs of purchasing or synthesizing millions of compounds. This problem is most noticeable for certain compound groups, such as metabolites, for which reference standards are often not commercially available. Due to the lack of reference standards, these compounds are often not included in mass spectral libraries unless scientists actively compile high-quality libraries [10]. Consequently, mass spectral libraries only cover a limited amount of chemical space, restricting the use of mass spectral libraries for compound identification. Additionally, identification results obtained with spectral libraries are strongly influenced by instrument type (analyzer and collision cell) and instrument settings, such as precursor isolation width, and by spectral curation steps, such as noise removal [11,12]. 

Multiple software tools have been developed to aid in the identification of compounds not included in mass spectral libraries [4,13]. These software tools use in silico predicted MS^2^ spectra to identify compounds instead of matching the MS^2^ spectra against a mass spectral library. In silico mass spectrum prediction is performed using molecular fingerprinting, rule-based fragmentation prediction, artificial intelligence, or a combination of these techniques. The experimentally obtained MS^2^ spectra that the user imports in these software tools are matched to the in silico MS^2^ spectra. Examples of these tools are CFM-ID [14], Chemdistiller [15], and MSfinder [16]. CFM-ID utilizes hybrid machine learning and rule-based fragmentation prediction. Chemdistiller is based on structural fingerprints and a machine learning algorithm. MSfinder uses rule-based in silico fragmentation prediction using hydrogen rearrangement rules.

Identification software tools are commonly evaluated using experimental MS^2^ spectra of chemical reference standards in a solvent and often use data extracted from curated mass spectral libraries. These spectra are less complex than MS^2^ spectra of the same compounds in matrix extracts. Moreover, differences can be observed in MS^2^ spectra obtained by different acquisition modes. The most commonly used acquisition mode for HR-MS^2^ spectra is a data-dependent top-n strategy (DDA), where the highest intensity m/z in a spectrum is selected for MS^2^ acquisition. This strategy yields MS^2^ spectra containing few interferences, which makes them relatively easy to interpret. However, the downside of this approach is that MS^2^ spectra for the compounds that fall outside the top-n are not acquired, making compound identification for low abundant signals impossible. To avoid this problem, data-independent acquisition (DIA) can be used. In DIA, MS^2^ acquisition is performed in parallel for co-eluting ions in a selected m/z range and merged into one fragment spectrum. In this way, DIA fragmentation spectra contain a composite of several co-eluting compounds and, therefore, are more challenging to match to spectral libraries or in silico predicted spectra.

Little is known about how different software tools compare in identifying compounds in solvent standards and spike into complex matrix extracts, especially for DIA spectra. In this study, we evaluated the capabilities of the software tools CFM-ID, Chemdistiller, MSfinder, and spectral library mzCloud for compound identification. The identification capability of each software tool using HRMS^2^ spectra of 32 compounds in solvent standards and the same compounds spiked into complex feed extracts acquired in DDA and DIA mode were assessed, and the obtained success rates were compared.

## 2. Materials and Methods

### 2.1. Chemicals

ULC grade methanol, acetonitrile, and water were obtained from Actu-All Chemicals (Oss, The Netherlands). Formic acid, acetic acid, and sodium acetate were obtained from VWR International (Darmstadt, Germany). Magnesium sulphate was obtained from Sigma-Aldrich (St. Louis, MO, USA). Reference standards were purchased from HPC Standards (Borsdorf, Germany), Sigma Aldrich, Witega (Berlin, Germany), and Santa Cruz (Dallas, TX, USA). To challenge the software tools, some of the selected compounds have the same molecular formula and very similar MS^2^ spectra. 

### 2.2. Solvent Standard and Spiked Feed Extract Preparation

The solvent standards were analyzed as three mix solutions (A, B, and C; Table 1). The mix solutions were prepared in-house from individual stock standard solutions and diluted in methanol to the desired concentration. The compounds were divided between the three mix solutions to avoid the inclusion of co-eluting compounds of the same molecular formula in one mix. Together, mix A, B, and C contained 32 veterinary drugs and pesticides in varying concentrations (40–2000 µg/L). The concentration of each compound was chosen to reflect the maximum residue limit and to ensure good detectability. The compound selection, including concentrations for each compound, is listed in Table 1. Only the high-concentration level data was evaluated for solvent standards. 

Animal feed, a compound feed for poultry, was extracted using a QuEChERS-based method. A total of 5 mL of water and 10 mL 1% acetic acid in acetonitrile was added to 5 g of animal feed. After extraction by shaking end-over-end at 50 rpm for 30 min, 1 g sodium acetate and 4 g magnesium sulfate were added to induce phase separation. The extracts were then centrifuged for 5 min at 3500 rpm. For the high concentration level, three 0.5 mL aliquots of the organic layer were transferred to clean tubes, and 0.5 mL of mix standard solutions A, B, and C were added to the 3 separate tubes. After evaporation to dryness, the extracts were reconstituted in 0.5 mL 50% methanol and transferred to an LC vial. For the low concentration level, three 0.5 mL aliquots were transferred to clean tubes, and 0.25 mL of mix standard solutions A, B, and C were added to the three tubes. After evaporation to dryness, the extracts were reconstituted in 500 µL 50% methanol and transferred to an LC vial. 

### 2.3. Instrumental Analysis

Liquid chromatography–high-resolution mass spectrometry (LC-HRMS) analysis was performed by using an Ultimate 3000 UHPLC system that was coupled to a Q-Exactive Orbitrap^TM^ system with HESI-II electrospray source (Thermo Scientific, San Jose, CA, USA). The system was controlled by using the software packages Xcalibur 4.3, Chromeleon 7.2.9, and Q-Exactive Tune 2.11. The instrument was calibrated before every analysis sequence with a maximum mass deviation of 1 ppm using Pierce LTQ ESI positive ion calibration solution (Thermo Scientific). Chromatographic and overall system performance was checked by analyzing a standard solution of 5 compounds before analysis and comparing mass accuracy, retention time, and intensity with previous performance test data. 

For DIA and DDA, the same chromatography was used. The eluents for the LC separation were (A) water and (B) methanol:water 95:5 (*v*/*v*), both containing 2 mM ammonium formate and 20 µL formic acid per liter. The LC flow rate was 300 µL min^−1^. The following gradient was used: 0% B for the first 0.1 min, linear to 45% B in 1.9 min, followed by a rise to 100% B in 6 min. After 6 min of 100% B, a switch back to 0% B was performed in 0.5 min before equilibration at 0% B for 4.5 min. The injection volume was 5 µL. The chromatographic separation was performed on an Atlantis T3 analytical column (100 mm × 3 mm, 3 µm particles, Waters, Milford, MA, USA) at a column temperature of 40 °C.

**Figure 1:**
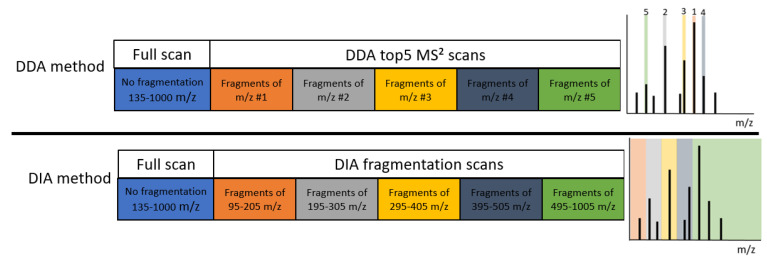
DDA and DIA acquisition methods visualized. The scans in each acquisition method, left, and on the right, the representation of the MS^1^ spectrum, where the coloring reflects the selections for the fragmentation scans. The DDA method (top) consists of a full scan measurement with a range of 135–1000 m/z, followed by acquisition of 5 consecutive data dependent MS^2^ spectra of the 5 signals with the highest intensity in the full scan MS^1^. The DIA method (bottom) consists of a full scan measurement with a range of 135–1000 m/z followed by the acquisition of 5 consecutive data independent fragmentation spectra of precursor mass ranges 95–205 m/z, 195–305 m/z, 295–405 m/z, 395–505 m/z, 495–1005 m/z.

Solvent standards and spiked feed extracts were analyzed in DIA and DDA mode. The DIA method was previously described by Zomer et al. [17]. In the DIA method, a total of 6 scan events were combined: 1 full scan event with a resolving power of 70,000 (defined at m/z 200, FWHM) for m/z range 135–1000 and 5 DIA fragmentation events with a resolving power of 35,000. The fragment scan events selected the precursor ion ranges m/z 95–205, 195–305, 295–405, 395–505, and 495–1005 for simultaneous fragmentation. DDA was performed by combining 2 scan events: 1 full scan event (mass range m/z 135–1000) with a resolving power of 70,000 (defined at m/z 200, FWHM) and a top 5-MS^2^ event with a resolving power of 17,500 (defined at m/z 200, FWHM) using a dynamic exclusion time of 10 s. In both DIA and DDA, the fragmentation was performed using higher-energy collisional dissociation (HCD) with a stepped collision energy of 30, 80 NCE. Figure 1 shows a visual representation of the DDA and DIA methods. 

### 2.4. Data Processing

Spectra were manually exported by using Xcalibur Qualbrowser version 4.5 (Thermo Scientific). For each MS^2^ spectrum, the top 20 highest intensity peaks were converted to the required file format for each tested software tool. DIA spectra were used without deconvolution. The experimental parent m/z and a 5-ppm mass tolerance was used as MS^1^ input. In all tools, all the available databases were used. Other settings were kept at default. The identification software tools were Chemdistiller version 0.1, MSFinder version 3.44, CFM-ID 3.0, and mzCloud. Both CFM-ID and mzCloud were accessed online between November 2020 and February 2021. The spectral library mzCloud contains multiple spectra of each compound generated with orbitrap analyzers. For most compounds, both HCD spectra and collision-induced dissociation (CID) spectra at multiple collision energies are available in mzCloud. In matching the experimental spectra to the library, no restriction on the type of collision cell or the collision energy matching tolerance was set. The other tools (Chemdistiller, MSFinder, CFM-ID) use in silico fragmentation instead of library spectra. Therefore, the type of analyzer, collision cell, and collision energy have limited effect on the identification results. We chose these tools to focus on single-step approaches that are easy to use and require limited expert knowledge.

The top three predicted identities and corresponding molecular formula for each measured spectrum were determined with all four software tools. If the correct compound was identified in the top 3 results, we considered the compound as identified correctly. It should be noted that some software tools repeat the same compound multiple times in the results for one spectrum based on multiple database sources. We strictly used the top 3 results, even if the top 3 contained the same compound identification multiple times.

## 3. Results

In Table 2, the percentages of correctly identified compounds are shown for DDA and DIA spectra for each software tool in the solvent standard and spiked feed extract. Three compounds in our test set were not included in the databases used by Chemdistiller. Therefore, two scores are given for this software tool: the score with the complete test set and the corrected score where the missing compounds are removed from the test set. 

For the DDA spectra of compounds in solvent standards, roughly 80% of the spectra were identified correctly. mzCloud has the highest identification score (84%). The other investigated software tools yielded results with slightly lower scores. 

The percentage of correctly identified compounds was slightly lower for the solvent standard DIA spectra compared to the DDA spectra, with an average of 69% compared to an average of 79%. This was expected since the DIA spectra are more complex compared to DDA spectra, although in solvent standards, this difference is less pronounced compared to spiked feed extracts, as solvent standards contain fewer interfering compounds. MSfinder and CFM-ID performed best with DIA spectra of solvent standards, with 72% correct identifications. Chemdistiller and mzCloud achieved 66% correct identifications in DIA spectra of solvent standards. 

Chemdistiller and mzCloud also perform less in identifying compounds from DIA spectra in spiked feed extracts. Especially at the lower evaluated concentration, identification success rates drop to 38% and 31%, respectively. The performance of MSfinder and CFM-ID in identifying compounds from DIA spectra in spiked feed extracts was higher than that of Chemdistiller and mzCloud but slightly lower than the performance in DDA spectra, with 72% and 63% correct identifications in spiked feed extract at the lower concentration. Lower scores in spiked feed extracts compared to solvent standards are expected due to the high complexity of the animal feed matrix. However, the results from MSfinder and CFM-ID seem less affected by these interferences than the other tools.

**Figure 2:**
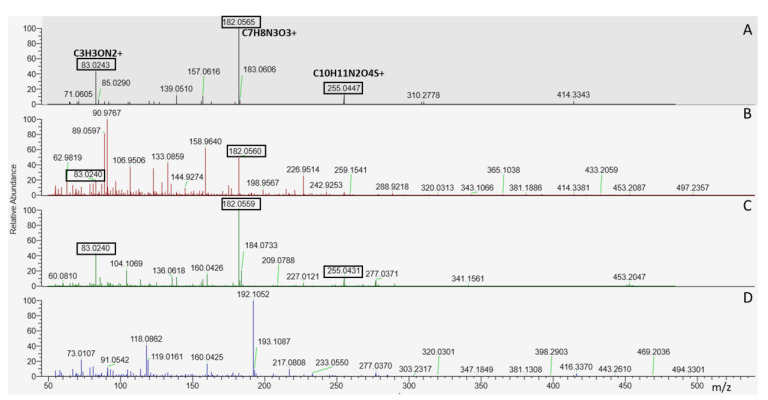
(**A**) DDA spectrum of foramsulfuron in solvent standard, (**B**) DIA spectrum of foramsulfuron in solvent standard, (**C**) DDA spectrum of foramsulfuron in animal feed extract, (**D**) DIA spectrum of foramsulfuron in animal feed extract. The annotated fragments are highly abundant in the DDA spectra but less abundant in the DIA spectrum in solvent and not visible in the DIA spectrum in animal feed matrix, where matrix interferences are abundant.

Lower performance in the spiked feed extracts (especially in DIA spectra) is mainly caused by matrix interferences, which are abundant in the DIA spectra and cause false identifications. Figure 2 shows an example of foramsulfuron. In the DDA spectra (A, C), foramsulfuron fragments C_3_H_3_ON_2_^+^, C_7_H_8_N_3_O_3_^+^, and C_10_H_11_N_2_O_4_S^+^ are the most abundant fragments. In the DIA spectra, these fragments are less abundant in solvent standard (B) and not visible in animal feed extract (D), where matrix interferences dominate the spectrum. Foramsulfuron was identified correctly in all software tools using DDA spectra but not in three of the tested software tools using DIA spectra. Only MSfinder correctly identified the DIA spectrum of foramsulfuron.

In Table 3, the results per compound are listed. The only compound correctly identified in all spectra (DDA and DIA in solvent and spiked feed extract) was sulfaguanidine, while ketoprofen and sulfadoxine were correctly identified in all spectra except one. However, some compounds were difficult to identify correctly, for example, sulfalene/sulfamonomethoxine and levoflocaxin/oflocaxin. These two sets of two included compounds are already difficult to distinguish in target acquisition and processing methods as they have the same m/z and retention time, and the most intense fragments have the same m/z. Several other compound sets (e.g., sulfamoxole/sulfisoxazole, doxycycline/epi-doxycycline, tetracycline/epi-tetracycline) are only distinguished by slightly different retention times (<0.5 min), not by m/z and most intense fragments. They can only be distinguished by low abundant fragments. These compounds are most frequently identified incorrectly by the evaluated software tools. 

Some other compounds also proved to be hard to identify, for example, cyproconazole. Cyproconazole was only identified correctly by CFM-ID, which provided correct identification in 5 out of 6 tested spectra, and by mzCloud in the DDA spectrum in solvent standard. The spectra of cyproconazole show only three fragments with a relative intensity >10%: the azole-fragment C_2_H_4_N_3_+, the fragment C_7_H_6_Cl+, and its ^37^chloride isotope. These fragments are common in azole-type pesticides. Therefore, the assignment of the correct compound within this compound class is complex.

On the other hand, all sulphonamides in the test set share multiple of the most abundant fragments. The sulphonamides included in the test set consist of three groups of two compounds each with the same molecular formula (sulfalene/sulfamonomethoxine, sulfamoxole/sulfisoxazole, sulfadimethoxine/sulfadoxine), which makes identification even more challenging. Still, as mentioned previously, most sulphonamides, except for sulfalene/sulfamonomethoxine, were correctly identified in both DDA and DIA spectra. The sulphonamides were present in a relatively high concentration in the solvent standard and spiked feed extracts. This increases the detection of lower abundance fragments needed for correct identification.

## 4. Discussion and Conclusions

Lately, DIA data acquisition approaches are increasingly used in untargeted analysis [18,19,20], as DIA can now be combined with DDA in one acquisition method. This approach is made possible by the latest-generation HRMS instruments, which have a much higher scan speed than earlier ones. However, research on small molecule compound identification with software tools using DIA data is unavailable. Therefore, this study evaluated the identification success rate of software tools CFM-ID, Chemdistiller, MSfinder, and spectral library mzCloud using DDA and DIA spectra of solvent standards and spiked feed extracts. 

The four evaluated software identification tools had a similar success rate in DDA spectra of solvent standards. Spectral library mzCloud provided the highest percentage (84%) of correct identifications in DDA spectra of solvent standards, followed by CFM-ID (81%). Our results applying in silico tools to DDA spectra are in line with results in the literature. The MSfinder release article [16] correctly identified 82.1% of the spectra in the top 3 of the results. In the CFM-ID 3.0 release article [14], the software correctly identified 93.3% of the spectra in the top 3 results. In the software comparison of Blazenovic et al. [21], in which the CASMI 2016 spectra of environmental xenobiotics and drugs were used, CFM-ID and MSfinder performed comparably, with 91.7% and 91.0% correct identifications in the top 5, respectively. Chemdistiller and mzCloud were not included in this comparison. Chemdistiller identified 86% of the test compounds correctly in the top 5 results in its release article [15]. To our knowledge, the identification success rate of mass spectral library mzCloud has not previously been studied. 

The results of this study show that identification using DIA spectra is more challenging for most software tools, especially in spiked feed extracts. Feed extract is a highly complex matrix, and very limited extract clean-up was performed. More clean-up of the extracts is not desirable in NTS as it can lead to the removal of compounds of interest. Despite the presence of matrix interferences, DIA spectra can be used for compound identification, with the highest success rates using MSfinder and CFM-ID. These two packages had similar identification scores for DIA and DDA spectra. Both software packages use in silico approaches for identification. It might be possible to improve the identification results in DIA spectra by applying spectral deconvolution prior to the use of the identification tools. In this study, we focused on easy to use, single-step approaches; therefore, we did not evaluate this option.

The conclusion of the first critical assessment of small molecule identification (CASMI) contest in 2013 was that spectral libraries provide better results than in silico tools [22]. However, in cases where the reference spectrum was not included in the library, in silico approaches and expert knowledge were required to obtain the correct identification. Our results show that mass spectral libraries and in silico approaches have a comparable success rate in DDA spectra. In contrast, the in silico approaches are more successful in compound identification for DIA spectra. 

In this study, only a limited number of compounds were included in the software evaluation. The compound selection mainly consisted of pesticides and veterinary drugs. These compound groups and their fragmentation patterns are well known and commonly used for scripting rules for in silico fragmentation prediction or as training data for machine learning approaches. Therefore, these compounds are easy to identify in automated approaches. However, our compound selection also included various challenges, for example, metabolites and multiple isomeric compounds. Additionally, we did not evaluate the identification success rate for combined approaches using multiple software tools. A combined approach may yield higher success rates, as described by Blazenovic et al. [21]. We focused on single-step approaches that are easy to use and require limited expert knowledge.

To summarize, this study demonstrates that in silico annotation tools and spectral library matching offer similar success rates in DDA spectra. DIA spectra can be used for compound annotation in a simple workflow without prior spectral deconvolution, and the rule-based approaches used by MSfinder and CFM-ID offer the best identification results out of the evaluated tools for DIA spectra, especially in complex matrix extracts.

## Figures and Tables

**Table 1:** Compound selection.

Name	Formula	High (µg/L)	Low (µg/L)	Mix
Albendazole-sulfone	C_12_H_15_N_3_O_4_S	2000	1000	A
Albendazole-sulfoxide	C_12_H_15_N_3_O_3_S	2000	1000	C
Clomazone	C_12_H_14_ClNO_2_	100	50	A
Cyproconazole	C_15_H_18_ClN_3_O	100	50	A
Doxycycline	C_22_H_24_N_2_O_8_	2000	1000	C
Epi-Doxycycline	C_22_H_24_N_2_O_8_	2000	1000	B
Fenbendazole-sulfoxide	C_15_H_13_N_3_O_3_S	200	100	A
Fenbufen	C_16_H_14_O_3_	500	250	A
Foramsulfuron	C_17_H_20_N_6_O_7_S	100	50	A
Indoprofen	C_17_H_15_NO_3_	500	250	C
Ketoprofen	C_16_H_14_O_3_	500	250	C
Levamisole	C_11_H_12_N_2_S	40	20	A
Levofloxacin	C_18_H_20_FN_3_O_4_	1000	500	B
Mebendazole-hydroxy	C_16_H_15_N_3_O_3_	40	20	C
Minocycline	C_23_H_27_N_3_O_7_	2000	1000	B
Naproxen	C_14_H_14_O_3_	200	100	A
Niflumic acid	C_13_H_9_F_3_N_2_O_2_	500	250	A
Ofloxacin	C_18_H_20_FN_3_O_4_	1000	500	C
Oxytetracycline	C_22_H_24_N_2_O_9_	2000	1000	C
Propyphenazone	C_14_H_18_N_2_O	500	250	C
Spinosyn-A	C_41_H_65_NO_10_	100	50	A
Sulfacetamide	C_8_H_10_N_2_O_3_S	2000	1000	A
Sulfadimethoxine	C_12_H_14_N_4_O_4_S	2000	1000	B
Sulfadoxine	C_12_H_14_N_4_O_4_S	2000	1000	C
Sulfaguanidine	C_7_H_10_N_4_O_2_S	2000	1000	C
Sulfalene	C_11_H_12_N_4_O_3_S	2000	1000	B
Sulfamonomethoxine	C_11_H_12_N_4_O_3_S	2000	1000	A
Sulfamoxole	C_11_H_13_N_3_O_3_S	2000	1000	A
Sulfisoxazole	C_11_H_13_N_3_O_3_S	2000	1000	A
Tetracycline	C_22_H_24_N_2_O_8_	2000	1000	B
Epi-tetracycline	C_22_H_24_N_2_O_8_	2000	1000	B
Tetramisole	C_11_H_12_N_2_S	40	20	C

**Table 2:** Percentage of correctly identified compounds in the top 3 for DDA and DIA spectra using the tested software tools for 32 compounds in solvent standards and spiked into animal feed extracts.

		Solvent Standard High Concentration	Spiked Feed Extract High Concentration	Spiked Feed Extract Low Concentration
DDA	MSfinder	75%	78%	81%
	CFM-ID	81%	81%	72%
	Chemdistiller	69% (76% *)	69% (76% *)	66% (72% *)
	mzCloud	84%	88%	84%
DIA	MSfinder	72%	75%	72%
	CFM-ID	72%	72%	63%
	Chemdistiller	59% (66% *)	47% (52% *)	34% (38% *)
	mzCloud	66%	44%	31%

* score corrected for three compounds missing in Chemdistiller databases.

**Table 3:** Identification results per compound. T: correct identification in top 3 results, F: no correct identification in top 3 results. (1) MSfinder, (2) CFM-ID, (3) Chemdistiller, (4) mzCloud.

	DDA												DIA											
	Solvent Standard				Spiked Feed Extracts				Spiked Feed Extracts				Solvent Standard				Spiked Feed Extracts				Spiked Feed Extracts			
	High Concentration				High Concentration				Low Concentration				High Concentration				High Concentration				Low Concentration			
	1	2	3	4	1	2	3	4	1	2	3	4	1	2	3	4	1	2	3	4	1	2	3	4
Albendazole sulfone	T	T	T	T	T	T	T	T	T	T	T	T	T	T	T	T	T	T	T	F	T	T	T	F
Albendazole sulfoxide	T	T	T	T	T	T	T	T	T	T	T	T	T	T	T	T	T	T	T	T	T	T	F	F
Clomazone	T	T	T	T	T	T	T	T	T	T	T	T	T	T	T	F	T	T	T	F	T	T	T	F
Cyproconazole	F	T	F	T	F	T	F	F	F	F	F	F	F	T	F	F	F	T	F	F	F	T	F	F
Doxycycline	T	T	T	T	T	T	T	T	T	T	F	T	T	F	F	F	T	T	F	F	T	T	F	F
epi-doxycycline *	F	F	F	F	F	F	F	F	F	F	F	F	F	F	F	F	F	F	F	F	F	F	F	F
epi-tetracycline *	F	F	F	F	F	F	F	T	F	F	F	T	F	T	F	T	F	F	F	F	F	F	F	F
Fenbendazole sulfoxide	F	T	T	T	T	T	T	T	T	T	T	T	T	F	T	T	T	T	T	F	F	F	T	F
Fenbufen	F	F	T	T	F	F	T	T	F	F	T	T	F	T	T	T	F	F	T	F	F	F	T	F
Foramsulfuron	T	T	T	T	T	T	T	T	T	T	T	T	F	F	T	F	T	F	F	F	T	F	F	F
Indoprofen	T	F	T	T	T	F	T	T	T	F	F	T	T	F	T	T	F	F	T	T	T	F	T	F
Ketoprofen	T	T	T	T	T	T	T	T	T	T	T	T	T	F	T	T	T	T	T	T	T	T	T	T
Levamisole	T	T	T	T	T	T	T	T	T	T	T	T	T	T	F	T	F	T	F	F	F	T	F	F
Levofloxacin	T	T	F	T	T	T	F	T	T	T	F	T	T	T	F	T	T	T	F	T	T	T	F	T
Mebendazole-hydroxy *	T	F	F	T	F	F	F	T	T	F	F	T	F	T	F	F	T	F	F	F	T	F	F	F
Minocycline	T	T	T	T	T	T	F	T	T	T	T	T	T	T	T	T	T	T	F	F	T	T	F	F
Naproxen	T	T	T	T	T	T	T	T	T	T	T	F	T	T	T	T	T	F	F	F	F	F	F	F
Niflumic acid	T	T	T	T	T	T	T	T	T	T	T	T	T	T	T	T	T	T	F	F	T	F	F	F
Ofloxacin	F	T	T	T	T	T	T	T	T	T	T	T	F	T	T	T	T	T	T	T	T	T	F	T
Oxytetracycline	T	T	T	T	T	T	T	T	T	T	T	T	T	F	T	T	T	F	F	F	T	T	F	F
Propyphenazone	F	T	F	T	T	T	T	T	T	T	T	T	F	F	F	T	T	T	T	T	T	T	T	T
Spinosin A	T	T	F	F	T	T	F	T	T	F	F	T	T	T	F	F	T	T	F	F	T	F	F	F
Sulfacetamide	T	T	T	F	T	T	T	T	T	T	T	T	T	T	T	F	T	T	T	T	T	T	T	F
Sulfadimethoxine	T	T	T	T	T	T	F	T	T	T	F	T	T	T	F	T	T	T	T	T	T	T	F	T
Sulfadoxine	T	T	F	T	T	T	T	T	T	T	T	T	T	T	T	T	T	T	T	T	T	T	T	T
Sulfaguanidine	T	T	T	T	T	T	T	T	T	T	T	T	T	T	T	T	T	T	T	T	T	T	T	T
Sulfalene	T	T	F	T	T	T	T	F	T	F	T	F	T	T	F	F	T	T	F	F	T	F	F	F
Sulfamonomethoxine	F	T	T	T	F	T	T	T	F	T	T	T	F	T	T	T	F	T	F	T	F	T	F	T
Sulfamoxole	T	T	T	T	T	T	T	T	T	T	T	T	T	T	T	T	T	T	T	T	T	T	F	T
Sulfisoxazole	T	T	F	T	T	T	F	T	T	T	F	T	T	T	F	F	T	T	F	T	T	T	F	T
Tetracycline	T	T	T	T	T	T	F	T	T	T	F	T	T	T	F	T	T	T	F	T	T	T	F	F
Tetramisole	T	F	T	F	F	F	T	F	F	F	T	F	T	F	T	F	F	F	T	F	F	F	T	F

* not included in Chemdistiller databases.

## Data Availability

Not applicable.

## References

[1] Pourchet M., Debrauwer L., Klanova J., Price E.J., Covaci A., Caballero-Casero N., Oberacher H., Lamoree M., Damont A., Fenaille F. (2020). Suspect and non-targeted screening of chemicals of emerging concern for human biomonitoring, environmental health studies and support to risk assessment: From promises to challenges and harmonisation issues. Environ. Int..

[2] Da Silva R.R., Dorrestein P.C., Quinn R.A. (2015). Illuminating the dark matter in metabolomics. Proc. Natl. Acad. Sci. USA.

[3] Theodoridis G., Gika H., Raftery D., Goodacre R., Plumb R.S., Wilson I.D. (2023). Ensuring Fact-Based Metabolite Identification in Liquid Chromatography–Mass Spectrometry-Based Metabolomics. Anal. Chem..

[4] Blaženović I., Kind T., Ji J., Fiehn O. (2018). Software tools and approaches for compound identification of LC-MS/MS data in metabolomics. Metabolites.

[5] Cai Y., Zhou Z., Zhu Z.-J. (2023). Advanced analytical and informatic strategies for metabolite annotation in untargeted metabolomics. TrAC Trends Anal. Chem..

[6] Kind T., Fiehn O. (2007). Seven Golden Rules for heuristic filtering of molecular formulas obtained by accurate mass spectrometry. BMC Bioinform..

[7] Trier X., van Leeuwen S.P.J., Brambilla G., Weber R., Webster T.F. (2023). Lack of chemical reference standards hinders (generation of) scientific evidence of chemical risks and their control. Environ. Health Perspect.

[8] Schymanski E.L., Jeon J., Gulde R., Fenner K., Ruff M., Singer H.P., Hollender J. (2014). Identifying Small Molecules via High Resolution Mass Spectrometry: Communicating Confidence. Environ. Sci. Technol..

[9] Thermo Scientific, mzCloud Advanced Mass Spectral Database. https://www.mzCloud.org.

[10] Bittremieux W., Wang M., Dorrestein P.C. (2022). The critical role that spectral libraries play in capturing the metabolomics community knowledge. Metabolomics.

[11] Kind T., Tsugawa H., Cajka T., Ma Y., Lai Z., Mehta S.S., Wohlgemuth G., Barupal D.K., Showalter M.R., Arita M. (2018). Identification of small molecules using accurate mass MS/MS search. Mass Spectrom. Rev..

[12] De Vijlder T., Valkenborg D., Lemière F., Romijn E.P., Laukens K., Cuyckens F. (2018). A tutorial in small molecule identification via electrospray ionization-mass spectrometry: The practical art of structural elucidation. Mass Spectrom. Rev..

[13] BMisra B.B. (2021). New software tools, databases, and resources in metabolomics: Updates from 2020. Metabolomics.

[14] Djoumbou-Feunang Y., Pon A., Karu N., Zheng J., Li C., Arndt D., Gautam M., Allen F., Wishart D.S. (2019). CFM-ID 3.0: Significantly Improved ESI-MS/MS Prediction and Compound Identification. Metabolites.

[15] Laponogov I., Sadawi N., Galea D., Mirnezami R., Veselkov K. (2018). ChemDistiller: An engine for metabolite annotation in mass spectrometry. Bioinformatics.

[16] Tsugawa H., Kind T., Nakabayashi R., Yukihira D., Tanaka W., Cajka T., Saito K., Fiehn O., Arita M. (2016). Hydrogen Rearrangement Rules: Computational MS/MS Fragmentation and Structure Elucidation Using MS-FINDER Software. Anal. Chem..

[17] Zomer P., Mol H.G. (2015). Simultaneous quantitative determination, identification and qualitative screening of pesticides in fruits and vegetables using LC-Q-Orbitrap™-MS. Food Addit. Contam. Part A.

[18] Guo J., Shen S., Xing S., Huan T. (2021). DaDIA: Hybridizing Data-Dependent and Data-Independent Acquisition Modes for Generating High-Quality Metabolomic Data. Anal. Chem..

[19] Hilaire P.B.S., Rousseau K., Seyer A., Dechaumet S., Damont A., Junot C., Fenaille F. (2020). Comparative Evaluation of Data Dependent and Data Independent Acquisition Workflows Implemented on an Orbitrap Fusion for Untargeted Metabolomics. Metabolites.

[20] Santos M.D., Camillo-Andrade A.C., Kurt L.U., Clasen M.A., Lyra E., Gozzo F.C., Batista M., Valente R.H., Brunoro G.V., Barbosa V.C. (2020). Mixed-Data Acquisition: Next-Generation Quantitative Proteomics Data Acquisition. J. Proteom..

[21] Blaženović I., Kind T., Torbašinović H., Obrenović S., Mehta S.S., Tsugawa H., Wermuth T., Schauer N., Jahn M., Biedendieck R. (2017). Comprehensive comparison of in silico MS/MS fragmentation tools of the CASMI contest: Database boosting is needed to achieve 93% accuracy. J. Chemin..

[22] Schymanski E.L., Neumann S. (2013). CASMI: And the winner is…. Metabolites.

